# List of Accidents, Admitted into the Pennsylvania Hospital, from January 10th to January 24th, 1838

**Published:** 1838-01-31

**Authors:** 


*List of Accidents, admitted into the Pennsylvania Hospital, from January 10th to January 24th,* 1838.

Two contusions of the side; one of the knee-joint, discharged cured in nine days. A luxation of the humerus downwards into the axilla, (reported above at length;) another luxation of the humerus, forwards under the pectoral muscle, of four weeks standing, the details and result of which will be given in our next. A wound from the bite of a dog, discharged on the day of admission, at his own request. A fracture of the fibula and tibia, extending into the knee-joint obliquely, accompanied with great effusion: V. S. ad ʃʒ℥xvii. a purgative, leeches to the part ad ʃʒ℥viii. low diet, cold lead water to be kept to the joint, and the limb to be placed in a long fracture-box. A fracture of the fibula, two inches above the ankle; one of the radius; and one of the ribs, complicated with a wound of the lung, and emphysema to the extent of five inches around the fracture, soon after the accident, accompanied with great oppression of the respiration and bloody sputa. A broad bandage was applied to the thorax V. S. on the second day, ʃʒ℥x; calomel gr. i. and; tartarized antimony gr. 1-8, every two hours, for two days, till the gums were touched. When this occurred, great relief was experienced. Dover's powder grs. viii. three times a day, and mucilage, with gruel diet. Doing well.

The lacerated wound of the hand, and the contusion of the abdomen, reported in our last, have been both cured. In the case of the gunshot wound of the hand, the sloughs all separated, and the man went out, labouring however under a slight attack of Erysipelas. The fractured clavicle, reported in No. 1, has been discharged, cured in 33 days; also one of the fractured fibulœ, cured in 27 days.

